# Health Literacy—Talking the Language of (School) Education

**DOI:** 10.3928/24748307-20190502-01

**Published:** 2019-07-19

**Authors:** Leena Paakkari, Orkan Okan

The links between school education and health have been of interest to researchers for several decades, and health literacy in particular has been associated with the health-promoting school approach for almost 20 years (St Leger, 2001; St Leger & Nutbeam, 2000). From an educational perspective, this is not surprising because there is little doubt that health literacy is a competence that contributes to health skill development and can be facilitated through educational practices. Although health-related skill development has been a core pillar of health promotion since the Ottawa Charter (Nutbeam, 1998), few countries have included health literacy as a theoretical framework within their school health curriculum (e.g., Australia [Australian Curriculum, Assessment and Reporting Authority, 2012], Finland (Finnish National Board of Education, 2014], and the United States [Joint Committee on Health Education Standards, 1995]), and even fewer have made the subject obligatory. As a result, few countries offer teacher training in relation to health literacy. This lack of training puts pupils as well as teachers in an unfavorable position because teachers are not equipped with health literacy teaching methods so pupils cannot be adequately supplied with health literacy skills.

Health literacy is strongly bound up with the field of education and forms a perfect bridge between the health and education fields. The benefits of including health literacy with education in the European Union have been shown in a recent policy brief (McDaid, 2016), and globally, health literacy was defined as an important education target at the World Health Organization's Shanghai conference in 2016 (World Health Organization, 2017). Therefore, in this present article, we wish to return to the discussion on linking education and health via health literacy, seeking common ground regarding the language used and the arguments presented. It may seem self-evident that when we talk about schools, we are talking about educational processes, but this is not always the case. We present three arguments that may bring new insights into the discussion and foster the uptake of health literacy in schools internationally.

## First Argument: Health Literacy Iis an Outcome of Education

Since its introduction in the 1970s (Simonds, 1974), health literacy has been particularly defined as a key outcome of health education (e.g., Nutbeam, 2000). However, researchers and politicians still appear to struggle with how health goals and health-enhancing practices can be linked to the practices of school education, or broadly speaking, how to break down the perceived division between health and education. As noted by Salganik (2006) during the 2006 Organisation for Economic Co-operation and Development (OECD) symposium in Copenhagen, people in the health fields often face the fact that “a connection between education and health is not a major element of [education] policy or ideology” and that “rationales for policies that support education generally do not include improving the health of the population” (p. 374). In line with available evidence on the subject, Salganik (2006) calls for the recognition of a link between school attendance and health. The learning of health-related skills in schools could pave the way for such a recognition early on in the life-course. According to the United Nations Educational, Scientific and Cultural Organization (2014), the purpose of education is to reduce inequalities. Given that health literacy explains disparities in health (e.g., Sun et al., 2013) and starts to develop in childhood, the development of health literacy corresponds well with the general aim of education. Also, health literacy is an educational phenomenon that can be developed and enhanced through educational practices, and it supports the gaining of academic competence together with civic and critical competence, in addition to the public health benefits it brings for both people and societies. As health citizenship and critical thinking have been defined as important health literacy dimensions (Nutbeam, 2000; Paakkari & Paakkari, 2012), facilitating and strengthening these skills via school health promotion is a goal that would perfectly merge the theoretical discussion surrounding health literacy with education practice. Furthermore, with the rise of online health information, society should respond to this challenge by providing schools with the necessary resources and infrastructure that allow the education system to offer children quality teaching regarding digital and e-health literacy skills.

Schools providing curriculum-based instruction offered by qualified teachers would seem to have advantages as compared to many other settings, especially when taking into account infrastructures and other conditions. Outside of schools, children from lower socioeconomic backgrounds and those suffering from poverty lack resources and opportunities to accumulate health literacy. Inside the school system, schools should respond to these inequities and create an environment that facilitates learning success for all children but more intensely for those in need and proportionally to the degree of their disadvantage, as suggested by Public Health England and the University College London`s Institute of Health Equity (Roberts, 2015). In this context, addressing the whole system to improve health literacy has been highlighted before (Brach et al., 2012), and this kind of systems thinking should be transferred to school health as well and be backed by polices that support schools to develop into schools that systematically promote health literacy and are able to respond to the health literacy needs of disadvantaged children.

## Second Argument: Health Literacy Corresponds Well with the Key Competencies Set Forth by the OECD

The Organisation for Economic Co-operation and Development (OEC) (2005) has discussed “key competencies” required in the modern world if people are to meet the demands placed on them by society. For a competence to be regarded as a key competence it has to “contribute to valued outcomes for societies and individuals; help individuals meet important demands in a wide variety of contexts; and be important not just for specialists but for all individuals” (p. 4). Drawing on this understanding, health literacy provides a good match with the OECD criteria for key competencies required by citizens, both cognitive and sociocultural. For example, health literacy covers abilities such as an ability to act in an ethically responsible way (Paakkari & Paakkari, 2012), empowers people to participate in promoting collective good (Nutbeam, 2000; Paakkari & Paakkari, 2012), and supports children and adolescents to critically analyze various media messages (Levin-Zamir, Lemish, & Gofin, 2011). Health literacy contributes to positive health outcomes, both at the individual and societal level, helps people to cope with and modify the factors that influence their own and others' health (McDaid, 2016; Nutbeam, 2000; World Health Organization, 2017), and therefore is relevant for every citizen. In addition, health literacy goes well beyond the basic skills of reading, writing, and numeracy; it is one of the conditions that must exist for people “to live a successful life and for society to face the challenges of the present and the future in modern, democratic societies” (Salganik & Provasnik, 2009, p. 255). Using the OECD perspective should serve as an example for the uptake of health literacy by nongovernmental organizations, who can start putting health literacy on their health policy agendas.

## Third Argument: Health Literacy Calls for Targeted Education on the Matter

The development of health literacy calls for a special kind of input. Although health literacy includes competencies (e.g., critical thinking and problem-solving skills) that are focused on many different school subjects, it cannot be guaranteed that the skills learned in school subjects will be applied to other contexts, such as the health context. As an example, Finnish students have performed well on The Programme for International Student Assessment (PISA); in 2015, PISA tested various skills and knowledge of 15-year-old students in 72 countries. Within certain subjects, the students have shown good competence in drawing conclusions and forming explanations. Nevertheless, they have shown only satisfactory competence in national assessments of health education, within which PISA-like skills were tested (Summanen, 2014). In assessing pupils' health-related competence (i.e., health literacy), it was found that the greatest challenges for pupils related precisely to these higher-order thinking skills. This mismatch could be an indication of a phenomenon familiar to experienced teachers; namely that skills are not necessarily transferrable. It may also suggest that high-level health literacy requires targeted time and resources allocated for health literacy instruction, including learning with a definite focus on higher-order thinking skills. In response, after the publication of the health education assessment findings, the health education curriculum was reformed, with new objectives for instruction and learning (Finnish National Board of Education, 2014). In the revised curriculum, the objectives are more demanding in terms of the skills required, and it was shaped to meet the challenges associated with health literacy that have been described throughout this article. As an example, in the current curriculum it is said, “pupils should be able to analyze the consequences of various ways of life on other people, and on the health of the environment,” a goal that was added to highlight the importance of sustainable and responsible behavior of the current and future generations (Finnish National Board of Education, 2014). The Finnish example could serve as a blueprint for other countries to develop and implement in their school health literacy curriculum.

## Conclusion

Health literacy fits well with education in schools, and school education can contribute to health by laying the foundations for health literacy in childhood and adolescence (World Health Organization, 2015). In accordance with the theorizing of Biesta (2010), it can be argued that health education in schools, among other social school goals, represents the prime purposes of education; namely qualification, socialization, and individuation. The incorporation of health literacy within the curriculum reflects the qualification function of education, which highlights the importance of developing the skills, knowledge, and understanding needed in various areas of life. The emphasis within health literacy on values such as participation, democracy, autonomy, responsibility, and sustainability reflects the socialization function, which broadly means that children can learn these values during educational processes. The individuation function, too, is manifested in health literacy, insofar as genuine health literacy involves pupils thinking critically, becoming aware of their own values and preferences, and finding their own voice, rather than passively following traditions. To us it is clear that when one is talking about health literacy in connection with schools, one is talking the language of education.

## References

[x24748307-20190502-01-bibr1] Australian Curriculum, Assessment and Reporting Authority. (2012). Shape of the Australian curriculum: Health and physical education. Retrieved from http://docs.acara.edu.au/resources/Shape_of_the_Australian_Curriculum_Health_and_Physical_Education.pdf

[x24748307-20190502-01-bibr2] Biesta G.J. (2010). Good education in an age of measurement: Ethics, politics, democracy. Boulder, CO: Paradigm Publisher.

[x24748307-20190502-01-bibr3] Brach C. Dreyer B. Schyve P. Hernandez L.M. Baur C. Lemerise A.J. Parker R. (2012). Attributes of a health literate organization. New York, NY: Institute of Medicine.

[x24748307-20190502-01-bibr4] Finnish National Board of Education. (2014). National core curriculum for basic education. Helsinki, Finland.

[x24748307-20190502-01-bibr5] Joint Committee on National Health Education Standards. (1995). National health education standards: Achieving health literacy. Retrieved from ERIC.gov website: http://files.eric.ed.gov/fulltext/ED386418.pdf

[x24748307-20190502-01-bibr6] Levin-Zamir D. Lemish D. Gofin R. (2011). Media health literacy (MHL): Development and measurement of the concept among adolescents. Health Education Research, 26(2), 323–335. 10.1093/her/cyr007 21422003

[x24748307-20190502-01-bibr7] McDaid D. (2016). Investing in health literacy: What do we know about the co-benefits to the education sector of actions targeted at children and young people? (Policy Brief No. 19). Copenhagen, Denmark: European Observatory on Health Systems and Policies, WHO Regional Office for Europe. 29144632

[x24748307-20190502-01-bibr8] Nutbeam D. (1998). Health promotion glossary. Health Promotion International, 13(4), 349–364. 10.1093/heapro/13.4.349 16963461

[x24748307-20190502-01-bibr9] Nutbeam D. (2000). Health literacy as a public health goal: a challenge for contemporary health education and communication strategies into the 21st century. Health Promotion International, 15(3), 259–267. 10.1093/heapro/15.3.259

[x24748307-20190502-01-bibr10] Organisation for Economic Co-operation and Development. (2005). The definition and selection of key competencies. Executive summary. Retrieved from https://www.oecd.org/pisa/35070367.pdf

[x24748307-20190502-01-bibr11] Paakkari L. Paakkari O. (2012). Health literacy as a learning outcome in schools. Health Education, 112(2), 133–152. 10.1108/09654281211203411

[x24748307-20190502-01-bibr12] Roberts J. (2015). Local action on health inequalities: Improving health literacy to reduce health inequalities. Retrieved from: Institute of Health Equity website: http://www.instituteofhealthequity.org/resources-reports/local-action-on-health-inequalities-health-literacy-to-reduce-health-inequalities

[x24748307-20190502-01-bibr13] Salganik L. (2006). Health behaviours: The competence approach. In: Desjardins R. Schuller T. (Eds.), Measuring the effects of education on health and civic engagement: Proceedings of the Copenhagen symposium (pp. 373–382). Copenhagen, Denmark: Organisation for Economic Co-operation and Development.

[x24748307-20190502-01-bibr14] Salganik L. H. Provasnik S. J. (2009). The challenge of defining a quality universal education: Mapping a common core. In: Cohen J. E. Malin M. B. (Eds.), International perspectives on the goals of universal basic and secondary education (pp. 252–286). New York, NY: Routledge.

[x24748307-20190502-01-bibr15] Simonds S. K. (1974). Health education as social policy. Health Education & Behavior, 2(Suppl. 1), 1–10. 10.1177/10901981740020S102

[x24748307-20190502-01-bibr16] St Leger L. (2001). Schools, health literacy and public health: Possibilities and challenges. Health Promotion International, 16(2), 197–205. 10.1093/heapro/16.2.197 11356758

[x24748307-20190502-01-bibr17] St Leger L. S. Nutbeam D. (2000). A model for mapping linkages between health and education agencies to improve school health. Journal of School Health, 70(2), 45–50. 10.1111/j.1746-1561.2000.tb07239.x 10715824

[x24748307-20190502-01-bibr18] Summanen A. M. (2014). Terveystiedon oppimistulokset perusopetuksen päättövaiheessa 2013. [The health education learning outcomes at the end of basic education in 2013 – English abstract]. Retrieved from Opetushallitus ja tekijä website: https://www.oph.fi/download/155889_terveystiedon_oppimistulokset_perusopetuksen_paattovaiheessa_2013.pdf

[x24748307-20190502-01-bibr19] Sun X. Shi Y. Zeng Q. Wang Y. Du W. Wei N. Chang C. (2013). Determinants of health literacy and health behavior regarding infectious respiratory diseases: A pathway model. BMC Public Health, 13(1), 261. . 10.1186/1471-2458-13-261 23521806 PMC3621712

[x24748307-20190502-01-bibr20] United Nations Educational, Scientific and Cultural Organization. (2014). UNESCO education strategy 2014–2021. Retrieved from https://unesdoc.unesco.org/ark:/48223/pf0000231288

[x24748307-20190502-01-bibr21] World Health Organization. (2015). Health 2020: Education and health through the life-course. Retrieved from http://www.euro.who.int/__data/assets/pdf_file/0007/324619/Health-2020-Education-and-health-through-the-life-course-en.pdf?ua=1

[x24748307-20190502-01-bibr22] World Health Organization. (2017). Shanghai declaration on promoting health in the 2030 Agenda for Sustainable Development. Health Promotion International, 32(1), 7–8. 10.1093/heapro/daw103 28180270

